# *Ex uno, plures*–From One Tissue to Many Cells: A Review of Single-Cell Transcriptomics in Cardiovascular Biology

**DOI:** 10.3390/ijms22042071

**Published:** 2021-02-19

**Authors:** Elvira Forte, Micheal A. McLellan, Daniel A. Skelly, Nadia A. Rosenthal

**Affiliations:** 1 The Jackson Laboratory, Bar Harbor, ME 04609, USA; Michael.Mclellan@jax.org (M.A.M.); dan.skelly@jax.org (D.A.S.); 2 Graduate School of Biomedical Sciences, Tufts University, Boston, MA 02111, USA; 3 National Heart and Lung Institute, Imperial College London, London W12 0NN, UK

**Keywords:** heart, cardiac, single-cell, scRNAseq, differentiation, development, injury, regeneration

## Abstract

Recent technological advances have revolutionized the study of tissue biology and garnered a greater appreciation for tissue complexity. In order to understand cardiac development, heart tissue homeostasis, and the effects of stress and injury on the cardiovascular system, it is essential to characterize the heart at high cellular resolution. Single-cell profiling provides a more precise definition of tissue composition, cell differentiation trajectories, and intercellular communication, compared to classical bulk approaches. Here, we aim to review how recent single-cell multi-omic studies have changed our understanding of cell dynamics during cardiac development, and in the healthy and diseased adult myocardium.

## 1. Introduction

The heart is our first functional organ. It starts beating very early in development, around E8.5 in mouse or 5–6 weeks of gestation in humans, and it beats about 3 billion times in an average human lifetime, pumping blood throughout the body to provide oxygen and nutrients while removing waste. This hard-working fluid pump function is achieved thanks to cardiomyocytes (CMs), which are highly specialized striated muscle cells, as well as a network of interstitial cells from vascular, nervous, immune, and mesenchymal/stromal lineages. Interstitial cells are responsible for building the proper 3D scaffold of the heart, ensuring proper CM alignment and synchronous beating. In response to injury, interstitial cells orchestrate the reparative response, leading to the scar formation, in order to compensate for the poor proliferative and regenerative capacity of CMs in adult mammals [1].

Gene expression studies are an accessible and cost-effective method for querying the molecular state of the heart during development, at homeostasis, or in response to insult. Until recently, these studies have largely been conducted either on cell populations enriched by cell sorting or on bulk cardiac tissue. Although both approaches have provided extremely valuable information, each is hampered by significant drawbacks. First, the main cellular constituents in cardiac physiology and disease have been defined based on the expression (or the lack thereof) of a limited number of surface antigens; thus, sorted cell populations may represent only a subset or a particular state within the cell population of interest [1]. Second, bulk tissue transcriptomics is biased toward the most prevalent cell types in the tissue, is confounded by compositional heterogeneity between samples, and may not represent the full spectrum of states within a cell population.

**Figure 1:**
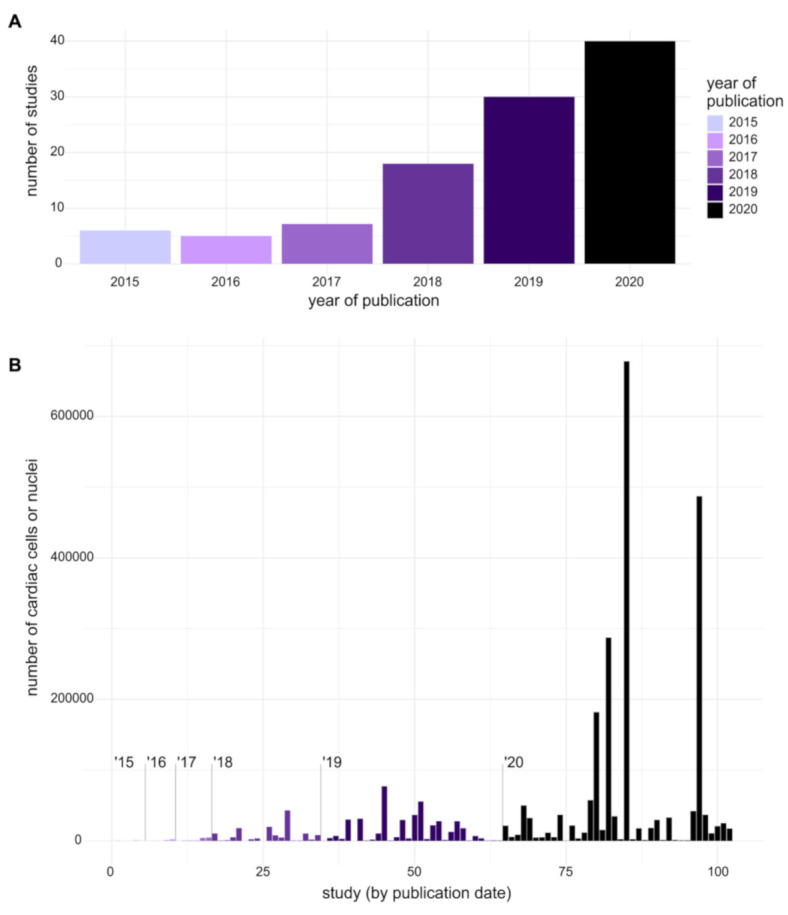
Overview of the exponential growth in single-cell transcriptomic studies from 2015 to 2020. (**A**) The number of published cardiovascular studies using single-cell transcriptomics per year. (**B**) The number of cells sequenced in each study. Studies in which the number of sampled cells could not be determined were omitted.

Single-cell RNA sequencing (scRNAseq) has emerged as a high-resolution alternative to bulk RNAseq and has gained popularity as a cardiovascular research tool (Figure 1A). Recent advances in scRNAseq technologies have enabled the profiling of individual cells in an unbiased fashion. Emerging methods for the analysis of scRNAseq data typically utilize a high-dimensional gene signature rather than any pre-selected list of canonical markers. These techniques have revealed significant cellular heterogeneity, paracrine inter-cellular communication among distinct cardiac cell populations, transcriptional kinships, potential intermediate states, and putative cellular differentiation trajectories.

The first attempts to sequence RNA from single cells were published almost 30 years ago and involved the manual microinjection of sequencing reagents into each disassociated cell [2,3,4]. Initial studies were limited to the analysis of a few genes by PCR. The development of integrated fluidic circuits (IFC) allowed for the quantification of the expression of multiple genes from the same cell, by reducing the reaction volume (Fluidigm Dynamic Arrays [5,6]). By optimizing cDNA library preparation, it became possible to capture whole-transcriptomes from single cells [7]. As of today, several scRNAseq protocols have been reported. One primary differentiating feature of these protocols is the method of cell isolation. These include methods that use nanofluidic traps (Fluidigm C1 IFC); the distribution of cells in wells by manual dilution, robotic dispersion, or FACS sorting (ICell8 Takara, Smart-seq2 [8], SORT-seq [9], Microwell-Seq [10], sci-RNA-seq3 [11]); and droplet-based systems (Drop-seq [12], sNucDrop-seq [13], InDrop [14]; Chromium Single Cells-10xGenomics [15]). The introduction of methods for automatic cell capture, lysis, and cDNA synthesis, together with the use of unique molecular identifiers (UMIs) [16] and molecular-based barcoding to multiplex cDNA amplification have contributed to plummeting per-cell costs, as evidenced by the exponentially increasing number of analyzed cells per study (Figure 1B). Compared to plate-based full-length sequencing [8], tag-based methods [9,10,11,12,13,14,15,16] detect fewer genes and capture only the 3′ or 5′ end of each transcript; however, the presence of UMIs improves gene-level quantification and mitigates biases in cDNA amplification. Moreover, new barcoding systems are allowing researchers to combine transcriptomic profiles with additional information from individual cells. For example, using polyadenylated DNA barcodes conjugated to antibodies, it is possible to estimate the abundance of specific surface antigens [17,18]. For an in-depth analysis of the history of scRNAseq and the comparison among different methods, we refer to [19,20,21,22].

Here, we review how single-cell technologies have impacted the cardiovascular field by allowing for high-resolution mapping of the heart at different stages of development and in pathological conditions, providing new insights on intercellular communication, cellular dynamic changes, and regulatory networks, as well as highlighting new potential therapeutic targets and prognostic biomarkers for patient stratification. Finally, we address the current limitations and future directions of the single-cell revolution.

## 2. Cardiac scRNAseq during Embryonic and Postnatal Development in Physiology and Disease

In the cardiac context, developmental biology and stem cell biology were the first fields to benefit from emerging single-cell transcriptomic techniques, which were used to define the cell fate and lineage trajectories in the developing heart and in vitro differentiating cells (Table 1). Two studies in 2015 adopted manual cell lysis and cDNA preparation combined with targeted single-cell qPCR (sc-qPCR) to analyze the early stages of cardiovascular commitment. Li et al. [23] first defined a panel of genes to distinguish all the main cardiovascular lineages, and they applied it to compare embryo- and mouse embryonic stem cell (mESC)-derived cardiac progenitor (CPs) and CMs at different stages; then, using time-lapse microscopy and multiplex sc-qPCR, they showed that while mESC-derived Nkx2-5^+^ CPs are bipotent and can become either CMs or smooth muscle cells (SMCs), Nkx2-5^+^ CPs in the embryos include two distinct unipotent subsets that differentiate either into endothelial cells (ECs) or CMs. Kokkinopoulos et al. [24] used a similar approach to profile about a thousand cells obtained from microdissected heart-forming regions from the early allontoic bud stage (EB, E6) to the early headfold stage (EHF, E8). After classifying the single-cell cDNA preparation chronologically, based on the expression of key markers, they selected 12 cells across four stages for deep sequencing, and the authors conclude that in the First Heart Field (FHF), Tbx5^+^ CPs exist transiently in the progenitor stage and rapidly differentiate post-activation of Nkx2-5 expression, after the EHF stage. One year later, two landmark papers provided the first transcriptomic maps of the developing hearts on multiple time points, using a semi-automated system, the Fluidigm C1 Integrated fluidic circuits (IFC), which allows for the capture, lysis, and retrotranscription of up to 96 single cells simultaneously [25,26]. The first study [25] profiled an unbiased selection of about 1200 cells from seven time points spanning from the early heart tube stage to the post-natal heart (E9.5, E11.5, E14.5, E18.5, P0, P3, and p21). The authors used this dataset as a reference to identify the developmental ages of mouse and human ESCs and to investigate the effect of the heterozygous mutations in *Nkx2-5* on lineage-specific differentiation. The second study [26] focused on three time points (E8.5, E9.5, E10.5), carefully dissecting two, seven, and nine regions, respectively. Combining transcriptomic and spatial information, they generated an algorithm to predict the anatomic origin of single CMs and the distribution of SHF Isl1^+^ derived cells, as well as to detect blocks in differentiation based on transcriptomic data. For example, when applied to *Nkx2-5^-/-^* CMs from E9.5 embryos, the algorithm revealed that a persistent atrial-like phenotype underlies the lack of ventricle development. The same technology has subsequently been used to selectively profile αMHC^+^ CMs in early and late development and post-natal hearts [27].

Additional studies have used the combination of lineage tracing, tissue microdissection, and scRNAseq to investigate the differentiation trajectory of CPs from the FHF and SHF [28,29,30]. Jia et al. [28] isolated CPs from *Nkx2-5-emGFP* transgenic and *Isl1^nGFP/+^* knockin embryos at E7.5, E8.5, and E9.5 and through scRNAseq, using Fluidigm C1 IFCs, and snATACseq (single nucleus assay for transposase-accessible chromatin with sequencing), they confirmed that Isl1^+^ CPs pass through an intermediate state before separating into different fates, whereas Nkx2-5^+^ CPs are unidirectionally committed to CM differentiation. Similarly, in a later study [30], the authors used *Isl1Cre:Rosa26^tdtomato/+^* and *Nkx2-5Cre::Rosa26^tdtomato/+^* embryos to isolate cells derived from the two lineages at E8.25, 8.75, 9.25, and they performed scRNAseq by Smart-seq2 [8] on manually isolated cells. They confirmed the previously identified pattern of differentiation of FHF and SHF CPs, and through ligand–receptor analysis, the authors suggest the *Mif*–*Cxcr2* pair as a possible mediator of the SHF CPs chemotaxis guided by CMs, which is a result that was confirmed by pharmacological inhibition and genetic knockouts of both *Cxcr2/Cxcr4*. Similarly, lineage tracing combined with Smart-seq2 has been used to analyze the arterial specification in the sinus venosus [31] and epicardial development in zebrafish [32].

Genetic knockouts, lineage tracing, and scRNAseq have been used in combination to define the early stage of cardiovascular lineage segregation from Mesp1^+^ mesodermal precursors [33], the role of the Hippo pathway [34], *Pitx2* [35], and *pbx4* [36] in development, the effect of *Hand2os1* lncRNA [37], and full microRNA KO [38]. The first study, similarly to what was described earlier [39,40], showed that Mesp1^+^ cells commit very early to different cardiovascular lineages, and the transcriptomic profiling at a single-cell level allowed for the identification of early pathways, determining the commitment to different cell fates, such as *Notch1* for the endocardium. Furthermore, by comparing *Mesp1* wt and null cells at early gastrulation (E6.75), the authors showed that *Mesp1* expression is required to exit the pluripotent state.

The second study used epicardial specific conditional knockout of the kinases Lats1/2 to interrogate the role of Hippo signaling in the commitment and differentiation of epicardial-derived cells. The authors profiled cells from wt and KO embryos at E13.5 and 14.5 before the appearance of cardiac phenotypes in the mutant using a high-throughput droplet-based system, Drop-seq [12], and they observed that mutant cells failed to differentiate in fibroblasts, maintaining an intermediate state with the expression of both epicardial and fibroblast markers. Additional pharmacological and genetic validation confirmed that an absence of Lats1/2 increased the nuclear localization of Yap, which inhibited fibroblast differentiation.

The third study [35] used scRNAseq in murine E10.5 and E13.5 wt and null embryos to analyze the molecular and cellular consequences of *Pitx2* disruption. *Pitx2* is a homeobox transcription factor, with pleiotropic functions during development. It is involved in OFT and valve development, left-right specification of the atria, and its loss has been implicated in human atrial fibrillation [41,42]. Consistent with previous work, the authors noted an altered cardiac cell composition and transcriptional changes in SHF progenitors that could lead to the prevalent differentiation in RA and sinoatrial node CMs.

A fourth study focused on Pbx4, a transcription factor similarly involved in SHF specification in zebrafish [36]. Single-cell transcriptomic analysis of Pbx4-depleted SHF progenitors showed a lack of heterogeneity and increased proliferation, which was in line with the cardiac dilation phenotype, and supporting its crucial role in the definition of the anterior and posterior progenitor fields that contribute to OFT and pharyngeal arches, respectively. In a fifth study [37], the authors showed that lncRNA *Hand2os1* strictly regulates *Hand2* expression in mouse; its full knockout caused a slight increase of *Hand2* in all cell types, which was sufficient to induce changes in cell composition and cell-specific transcriptome, leading to morphological and functional abnormalities. In a sixth study [38], full microRNA KO in CMs was achieved by conditionally deleting the microprocessor *Dgcr8* in Mesp1^+^ cells. As a result, the heart appeared dilated and the CMs upregulated vascular genes, demonstrating that microRNAs are required to suppress the angiogenesis program and allow CM differentiation.

Unbiased profiling of microdissected regions and paired-scRNAseq by Smart-seq2 have been used to define early mesoderm patterning during gastrulation (E6.5–7) [43], and more recently, to generate a highly time- and space-resolved profile of the mouse anterior cardiac region from the cardiac crescent to the linear tube stage [44]. For this latter study, the author collected samples at six time points, 12 h apart (E7.75–8.25), identified six cardiac clusters at different levels of differentiation, and assigned them to specific locations of the developing heart using scRNAseq data from four dissected regions of the cardiac mesoderm as a spatial reference. Based on gene expression and localization, the sub-populations were attributed to SHF or FHF, and differentiation trajectories, inferred on transcriptomic statuses, were validated by lineage tracing combined with single-cell resolution microscopy and in situ hybridization chain reaction. These analyses led to the identification of an extra progenitor field, anterior to the cardiac crescent, which was named the juxta-cardiac field (JFC). These *Nkx2-5*^−^ *Hand1*^+^ *Mab21l2*^+^ cells are derived from *Mesp1*^+^ progenitors and contribute to both the proepicardium and the myocardium.

Microdissection and high-throughput scRNAseq, using the 10x Chromium technology, enabled the characterization of the murine cardiac outflow tract development in early, middle, and late stages of remodeling and septation (47-, 49-, 51- pairs of somites, E11-12) [45], as well as of the wild-type murine conduction system (at E16.5) [46], providing reference transcriptomic maps to compare normal and altered conditions.

A few studies have aimed to define atlases of the full cardiac embryonic development. In the same year, one study profiled about 4000 cells from different stages of human fetal heart development [47], and another defined a Mouse Organogenic Cell Atlas (MOCA), analyzing over 2,000,000 total cells from murine embryos from five different stages, including over 7000 cells of the cardiac lineage [11]. In the first study, the authors isolated cells from various regions of fetal hearts in early (5–7 weeks), mid (9–17 weeks), and end stage (20–25 weeks) of gestation. Cells were manually picked, lysed, and the RNA was retrotranscribed using a modified version of STRT-Seq (Single-Cell Tagged Reverse Transcription Sequencing) [16]. The authors identified four main cell clusters, defining their spatial distribution and progressive transcriptomic changes during development. Furthermore, the authors compared these data with previously published mouse embryonic datasets [26,34] to identify correspondent stages and highlight the common and unique markers of each cell type. For the second study, the authors used a different plate-based system, sci-RNA-seq3 [48], that utilizes combinatorial indexing to profile millions of cells in the same experiment. In this particular study, nuclei were isolated from snap-frozen murine embryos (E9.5, 10.5, 11.5, 12.5, and 13.5 embryos), profiling enough cells to cover 3–80% of a full embryo per stage. Despite shallow sequencing allowing the detection of only a few hundred transcripts per cell, the authors were able to identify over 500 cell subtypes and 56 developmental trajectories, which can be publicly explored online [49]. 

More recently, researchers have pioneered unbiased Spatial Transcriptomics [50] and combined it with in situ sequencing by single molecule fluorescence in situ hybridization (smFISH), and scRNAseq to recreate organ-wide cell type/gene expression maps of the developing human heart in three main stages: 4.5–5, 6.5–7, and 9 weeks post conception [51]. These data can be explored both in 2D and 3D on a public web platform [52]. 

One potential use of these publicly available atlases is to provide a baseline framework for comparison to congenital diseases and abnormal conditions. At least three studies in the past three years have used scRNAseq to investigate congenital heart diseases. Hu et al. [53] used a droplet microfluidic-based snRNA-seq method (sNucDrop-seq [13]) to unbiasedly profile cells from post-natal hearts of wild-type (P6 and p10) and ERRα/γ knockout mice (p10) [54], creating a model of pediatric mitochondrial cardiomyopathy. They observed cell-specific gene expression changes, with CMs, fibroblasts, and ECs being the most affected cell types. Most of the cells presented a downregulation of genes related to oxidative phosphorylation, and an upregulation of fibrosis genes was observed also in non-fibroblasts. Furthermore, single-cell transcriptomic data revealed that the expression of Gdf15, a cardiac-produced hormone and marker of cardiac dysfunction, was regulated by distinct cell-type-specific networks. More recently, two studies have used 10x Chromium technologies to investigate different models of congenital heart defects [55,56,57,58]. The first study [55] profiled the cardiogenic region of wild-type versus *Hand2 null* mouse embryos over three phases of cardiac development (E7.75, E8.5, E9.25). The *Hand2* deletion causes embryonic lethality by E10.5, in the absence of right ventricle development [59]. Temporal transcriptomic analysis revealed that RV precursors were indeed specified but failed to properly migrate and differentiate, while OFT specification was impaired, thus providing new insights on the cellular mechanisms of the altered development. A case of autoimmune-mediated congenital heart block (CHB) in a 21-week-old human fetus has been analyzed by profiling cardiac cells from the affected fetus and cells from control fetuses at 19–22 weeks of gestation [57]. CHB affects mid-gestation normal developing fetuses that have been exposed to maternal autoantibodies. Differential gene expression revealed an increase and heterogenous IFN response in different cell types of the affected heart and the expression of ECM genes, which is in line with the observed fibrosis in the AV node.

In summary, single-cell transcriptomics has facilitated advances in several areas of developmental biology: the derivation of spatial and temporal maps of embryonic cardiac morphogenesis, the identification of new progenitor fields without the restraints of known marker genes, and the inference of cell–cell interactions and differentiation trajectories, which can then be validated with classical approaches such as lineage tracing or gene knockdown. These high-resolution road maps can be used as a reference to identify cell-specific transcriptional changes in congenital heart diseases as well as potentially revealing novel therapeutic targets.

**Table 1:** Cardiac Development in Physiology and Disease.

Authors PMID	Date	# of Cells and/or Nuclei	Isolation Method	Sequencing Technology	Target Cell Types	Context
Li et al. [23] PMID: 25633351	January 2015	448 cells	enzymatic digestion, FACS	Manual cell lysis/cDNA preparation; targeted sc-qPCR Fluidigm Dynamic Array IFCs [6]	CMs, ECs, CFs, SMCs	Comparison of embryo (E10.5)- and mESC-derived cardiac progenitor and CM differentiation
Kokkinopoulos et al. [24] PMID: 26469858	October 2015	1088, 12 deep- sequenced	enzymatic digestion	Manual cell lysis/cDNA preparation; targeted Taqman^®^ sc-qPCR; Illumina GA IIx	Cells from the heart forming regions in early mouse embryos	Profile the early FHF cardiac progenitors. Early EB to EHF stage (E6-8)
DeLaughter et al. [25] PMID: 27840107	November 2016	≈1200	enzymatic digestion	Fluidigm C1 IFCs; Illumina HiSeq2500	embryonic and post-natal cardiac cells	Embryonic to postnatal development: E9.5, E11.5, E14.5, E18.5, P0, P3, p21 and comparison with differentiating mESC and hESC
Li et al. [26] PMID: 27840109	November 2016	2233	enzymatic digestion	Fluidigm C1 IFCs, Illumina HiSeq2000; targeted sc-qPCR Fluidigm 96x96 Dynamic Array	embryonic cardiac cells	Early murine embryo development: E8.5, E9.5, or E10.5 hearts dissected in multiple zones
Lescroart et al. [33] PMID: 29371425	January 2018	672	enzymatic digestion, FACS in lysis buffer	Smart-seq2[8]; Illumina Hi-Seq 2500	Mouse embryonic derived cardiac progenitor cells (Mesp1^+^)	Early stage of cardiovascular lineage segregation: Mesp1^+^ progenitor from wt and Mesp1 null embryos E6.75-7.25
Xiao et al. [34] PMID: 29689192	April 2018	18,166	enzymatic digestion	Drop-seq [12]; Illumina Nextseq500	embryonic cardiac cells	Role of Hippo signaling in murine embryo development. CTR versus Lats1/2 CKO E13.5 and E14.5 embryos
Sereti et al. [27] PMID: 29467410	April 2018	122	mechanical and enzymatic digestion, FACS	Fluidigm C1 IFCs; Illumina NextSeq 500	αMHC^+^ (αMHC-GFP)	CM heterogeneity in E9.5, E12.5, and P1 mouse hearts
Su et al. [31] PMID: 29973725	July 2018	2384	mechanical and enzymatic digestion, FACS	SMART-seq2, Illumina Nextera XT, NextSeq500	*ApjCreER* labeled SV-cells (E12.5–14.5), and Coup-tf2^OE^- SV cells (E14.5)	Coronary artery specification in the SV
Chen et al. [38] PMID: 30128894	August 2018	152	mechanical and enzymatic digestion, mouth pipette	Smart-seq2; Illumina Hi-Seq 4000	ventricle from E9.5 heart tube, wt and *Mesp1^Cre/+^ x Dgcr8^loxp/loxp^*	Effect of global microRNA KO on cardiac development
Hu et al. [53] PMID: 30254108	September2018	≈20,000	mechanical nuclei isolation	sNucDrop-seq [13]	early post-natal cardiac cell nuclei	Postnatal heart development in WT (p6, p10) and ERRα/γ knockout mice (p10, pediatric mitochondrial cardiomyopathy)
Jia et al. [28] PMID: 30451828	November 2018	421	enzymatic digestion, FACS	Fluidigm C1 IFCs, ICELL8™ Single-Cell System (Wafergen); Illumina NextSeq 500	cardiac progenitor cells from Nkx2-5-emGFP and Isl1^nGFP/+^ embryos	CPs developmentE7.5, E8.5, and E9.5 embryos
Cui et al. [47] PMID: 30759401	February 2019	4000	enzymatic digestion, mouth-picking, FACS	Manual cell picking-lysis; STRT-seq [60]; paired-end sequencing Illumina 4000	human cardiac fetal cells (6, 7, 13, 17 wks); ECs 22 wks, VCs 17 wks	Spatial/temporal analysis of human cardiac development Comparison with mouse data
Cao and Spielmann et al. [11] PMID: 30787437	February 2019	7089-cardiac muscle lineage (2,058,652 total)	Nuclei from snap frozen embryos, no enzymatic digestion, dispersed in 96 well plates	In plate sci-RNA-seq3; NovaSeq (Illumina)	embryonic cells	Mouse organogenic cell atlas (MOCA) E9.5, E10.5, E11.5, E12.5, E13.5.
Li et al. [29] PMID: 31142541	June 2019	>10,500	enzymatic digestion	Chromium Single Cells 3’ v2 (10x Genomics); Fluidigm C1 IFCs, Illumina’s HiSeq 2500 and 4000	embryonic cardiac cells, *Isl1-cre/mTmG* embryos	Profiling ventricular chambers of E10.5 heart, by dissection and lineage tracing
Hill et al. [35] PMID: 31201182	June 2019	77,122	mechanical and enzymatic digestion	Chromium Single Cells 3’ v2 (10x Genomics); Illumina NextSeq 500	embryonic cardiac cells	E10.5 and E13.5, control and *Pitx2* mutant hearts
Han et al. [37] PMID: 31273086	July 2019	3600	mechanical and enzymatic digestion, FACS	Chromium Single Cells 3’ v2 (10x Genomics); Illumina HiSeq 2500 and HiSeq X TEN	embryonic cardiac cells wt and *Hand2os1*-null	Effect of the lncRNA *Hand2os1*- on cardiac development
Yvanka de Soysa et al. [55] PMID: 31341279	July 2019	36,654	Micro-dissection and enzymatic digestion	Chromium Single Cells 3’ v2 (10x Genomics); Illumina NextSeq 500 and HiSeq4000	embryonic cardiac cells	Effect of congenital mutation on cardiac development (Hand2-null versus wt, E7.75, E8.25, and E9.25 embryos)
Liu et al. [45] PMID: 31365875	July 2019	55,611	enzymatic digestion	Chromium Single Cells 3’ v2 (10x Genomics); Illumina NovaSeq 6000	embryonic cells from the OFT	Murine OFT development (ps47, ps49, ps51)
Xiong et al. [30] PMID: 31221018	August 2019	2631	Micro-dissection and enzymatic digestion, FACS	Smart-seq2; Illumina HiSeq 4000	embryonic cardiac cells	Differentiation trajectory and interlineage communication of cardiac progenitor cells from FHF and SHF (E8.25, 8.75, 9.25)
Goodyer et al. [46] PMID: 31284824	August 2019	>22,000	Micro-dissection and enzymatic digestion	Chromium Single Cells 3’ v2 (10x Genomics); Illumina HiSeq4000	embryonic cardiac cells	Cardiac conduction system in the embryo E16.5
Asp and Giacomello et al. [51] PMID: 31835037	December 2019	3717	Mechanical and enzymatic digestion, FACS in 384w plates	Chromium Single Cells 3’ v2 (10x Genomics); Illumina HiSeq2500	embryonic cardiac cells	Spatio-temporal transcriptomic of developing human heart at different stages
Weinberger et al. [32] PMID: 32084358	February 2020	at least 5000 cardiac B-cells	mechanical and enzymatic digestion, FACS	Smart-seq2 and TARGET-seq [61]; Illumina NextSeq500	fluorescent epicardial reporters (e.g., *tbx18:myr-*eGFP)	Epicardium heterogeneity in zebrafish cardiac development
Holowiecki et al. [36] PMID: 32094112	March 2020	5300	mechanical and enzymatic digestion, FACS	Chromium 10x; Illumina HiSeq2500	*nkx2.5*:ZsYellow^+^ cells at 28 hpf	*pbx4* depletion and OFT development in zebrafish
Suryawanshi et al. [57] PMID: 31589297	July 2020	17,747	Langendorff enzymatic perfusion	Chromium Single Cells 3’ v2 (10x Genomics); Illumina HiSeq2500	whole human fetal hearts	Congenital heart block (CHB): comparison of healthy versus anti-SSA/Ro-associated CHB foetal hearts (mid-gestation)

## 3. Cardiac scRNAseq to Elucidate In Vitro Differentiation and Reprogramming

Single-cell technologies have proven to be invaluable tools for defining cellular heterogeneity and lineage commitment in the context of in vitro cell reprogramming and differentiation (Table 2). At least four published studies have focused on the reprogramming of cardiac fibroblasts to induced CMs (iCM). The first [62] used sc-qPCR to study how to improve a seven-factor protocol to obtain iCMs from human fibroblasts [63]. The authors tested the protocol on hESC-derived hCFs and observed that no additional factor could increase the yield of iCMs; however, HAND2 or microRNA-1 could enhance the CM phenotype, as shown by comparison with hESC-derived CM. Soon after, a separate group adopted the Fluidigm C1 technology to profile 574 neonatal murine fibroblasts three days post-transfection with Mef2c, Gata4, and Tbx5 viruses [64]. The authors identified four main transcriptomic states, which were predicted to form a continuum in the transition to iCMs, and they validated their temporal occurrence with population-based gene expression analysis at six time points post-transfection. By differential gene expression analysis, they observed that the acquisition of CM-specific splicing patterns was a key process in the progressive trans-differentiation; and depletion of the splicing factor *Ptbp1* could increase reprogramming efficiency. The same group used a similar approach to profile the gene networks and cellular states underlying the more challenging conversion of human adult fibroblasts to iCM [65]. The authors inferred a trajectory to place cells along an axis of pseudotime, which is a measure of how far a cell has progressed along a biological process, which is predicted based on associations between transcriptomic states and characterized by continuous gradients in gene expression. The pseudotemporal trajectory revealed two branches with cells either differentiating to iCM or going back to fibroblasts, and genes involved in the immune response-associated methylation appeared to be crucial for the transition to iCM. Finally, a recent study combined scRNAseq, ATAC-seq, and ChIP-seq (chromatin immunoprecipitation followed by sequencing) for an in-depth profiling of the effect of Mef2c, Gata4, and Tbx5 expression (individual or combined) in embryonic murine fibroblasts reprogrammed to iCM [66].

The process of hiPSCs differentiation to CM has been initially profiled at multiple time points by two distinct studies [67,68], revealing regulators that can enhance CM differentiation [68], and the conditions that could lead to a prevalence of atrial-like versus ventricular-like CMs [67]. A later study selectively profiled hiPSC-derived CMs at the mid and late stage of differentiation (d12, d40), combining RNAseq data obtained by Fluidigm C1, with electrophysiological measurements obtained in a non-invasive way, by transfecting cells with a construct that allows the visualization of changes in polarization as changes in fluorescence intensity (via ArchLight) [69]. The authors concluded that gene expression was insufficient to predict the electrophysiological state of the CMs, but by differential expression between two stages of differentiation, they identified ion-channel regulators that modulate the hiPSC-CM maturation. In particular, genetic ablation of one of these regulators (*FHL1*) could lead to ventricular-like APs in the differentiating cells. Finally, a more recent study used the progressive and heterogeneous differentiation of hiPSCs to CMs to compare scRNAseq data obtained by Drop-seq versus DroNc-seq [70]. By analyzing in parallel cells from multiple time points, they observed six distinct populations, five of which were identified by both methods, and both datasets could be used to infer similar pseudo-temporal trajectories.

Further, scRNAseq has been employed to analyze patient-derived hiPSC and dissect the cellular basis of cardiomyopathy associated with Duchenne muscular dystrophy (DMD) [71] and the defective cardiac development in Hypoplastic Left Heart Syndrome (HLHS) [56] and a type of Hypoplastic Right Heart Syndrome (HRHS) [58]. DMD is the most common form of muscular dystrophy, it is caused by mutation in the *DMD* gene, and it generally leads to dilated cardiomyopathy (DCM). The scRNAseq analysis showed that hiPSC-CM from DMD patients presented a transcriptional dysregulation similar to what was observed in patients’ cardiac biopsies and mouse models of DMD, highlighting their utility as a model for drug testing. HLHS is a complex, multifactorial congenital heart disease [56]. To study its etiology, the authors first profiled cells isolated from different regions of a human fetal heart at 83 days of gestation and confirmed that the expression of genes previously reported as having *de novo* mutations associated with HLHS was higher in endothelial/endocardial clusters. Then, they used hiPSC-derived EC from healthy and HLHS patients to dissect the molecular basis of endocardial abnormalities, and with the use of in vitro functional assays, they showed that endocardial defects could lead to impaired EndMT and angiogenesis as well as reduced CM proliferation and maturation. HRHS is associated with pulmonary or tricuspid valve atresia, and it is prevalent in the Asian population. To probe a class of HRHS, Pulmonary Atresia with Intact Ventricular Septum syndrome, the authors profiled hiPSC-CM derived from three patients and three healthy controls, cultured in three different conditions: regular cultures, anisotropic organoids, and cardiac tissue strips [58]. The two bioengineered constructs are devised to measure electrophysiological and contractile responses, respectively, and promote CM maturation. The transcriptomic data on hiPSC-CM at different maturation stages revealed a downregulation of contractile and maturation genes in the HRHS patients and upregulation of immature transcripts, suggesting intrinsic defects in the CMs that could explain the limited RV growth even after interventions to establish the RV–pulmonary arterial connection.

Single-cell transcriptomics of differentiating ESCs has been used to profile the heterogeneity of Mesp1-induced mesodermal cells [72] and hESC-derived epicardial cells [73], to define the steps leading to CM differentiation [74], and to assess mESC-CM maturation post-in vivo transplantation [75]. The process of differentiation of human ESCs to CMs was analyzed by performing scRNAseq over six different stages using the iCell8 platform. The authors reconstructed pseudotemporal trajectory and identified putative ligand–receptor interactions. They observed a crosstalk between cardiac progenitor cells and endocardial cells at d5, leading to the activation of the transcription factor ETS1. By ChIP-Seq and genetic depletion, they showed that ETS1, and therefore the CPs–endocardial cell communication, is important for cardiac lineage commitment [74]. The same group used the ICell8 platform to dissect post-natal CM differentiation [76]. Integrating previously generated scRNAseq on LV cells at p1, p4, p7, and p14 with new data at p56, they identified fibroblasts as a crucial cell type promoting CM differentiation, which they confirmed in vitro with the co-culture of immature CM, isolated from p1 pups, with neonatal or adult fibroblasts. Single-cell analysis has been also used to analyze the reverse process of adult CM reprogramming to mCPs [77] as well as assess the negative effects of nicotine on hESC differentiating into CMs [78], proving to be a valid platform to determine embryonic toxicity; and to establish the best combination of transcription factors to obtain pacemaker-like cells from Nkx2-5^+^ CPs, which are derived from the transdifferentiation of adult human adipogenic MSCs (hASMSCs) [79].

Similar timeline studies of cellular differentiation are lacking for adult stem/progenitor cells (Table 3). Target sc-qPCR has been used to profile different subpopulations of human cardiac Lin-Sca1^+^ cells are defined based on the side population phenotype, the expression of CD31 or PDGFRa^+^, confirming that the Sca1^+^/PDGFRa^+^ fraction included the population of clonogenic progenitor cells [80]. An earlier paper analyzed the inverse process in mice, the de-differentiation of CMs to CMs-derived cardiac progenitor cells, using microarrays for single-cell transcriptomic profiling, sc-qPCR for validations, and microarrays for bulk DNA methylation analysis [77]. One of the main applications of stem/progenitor cells is cardiomyoplasty, involving transplantation in the infarcted myocardium to promote regeneration. At least two studies employed single-cell transcriptomic analysis to assess the paracrine function of progenitor cells injected in mice heart subjected to coronary ligation or sham-operated [81,82]. The first study analyzed bone marrow-derived mesenchymal stem cells, 10 days post-injection, the second analyzed hiPSC-derived CMs 4 days post-injection. Both studies combined bulk transcriptomic data on laser capture microdissected samples, with single-cell Taqman^®^ sc-qPCR through Fluidigm Dynamic Arrays, to show the beneficial factors secreted by the cells of interest, post-injection in the infarcted hearts. More recently, three studies have focused on the controversial population of c-kit^+^ progenitor cells [83,84,85]. In all cases, scRNAseq analysis showed that freshly isolated c-kit^+^ cells are heterogeneous and include cells with mesenchymal or endothelial features; thus, they are unlikely to differentiate in CMs. By lineage tracing and IHC, the first study suggested a minimal differentiation of c-kit^+^ cells to CM in response to trans aortic constriction (TAC), which was relatively enhanced in response to doxorubicin-induced cardiac injury [83]. The second study revealed that cultured c-kit^+^ cells lose the heterogeneity and identity markers of freshly isolated cells and include only two subclusters (expressing cell adhesion and metabolism-related genes, respectively), providing a possible explanation for the limited beneficial effect in clinics [84]. The same group highlighted another possible source of discrepancy between preclinical and clinical studies: the presence of tetraploid c-kit^+^ cells in rodents but not in pigs or humans [85]. Tetraploid cells escape the replicative senescence, and through scRNAseq, the authors observed that 4N cells tended to express endothelial-related genes, while 2N cells appeared more closely related to fibroblasts.

Overall, these studies show the essential role of single-cell transcriptomics in predicting the function and differentiation fate of stem/progenitor cells in normal conditions and in response to stressors such as injury stimuli, drug treatments, and genetic mutations. The identification of intermediate transcriptomic states provides novel insights into reprogramming and differentiation that are often difficult to interrogate by other means.

**Table 2:** In Vitro Cardiac Cell Differentiation- Embryonic and Pluripotent Stem Cells.

Authors PMID	Date	# of Cells and/or Nuclei	Isolation Method	Sequencing Technology	Target Cell Types	Context
Chan et al. [72] PMID: 27131741	April 2016	94	enzymatic digestion, FACS (live cells)	Fluidigm C1 IFCs; Illumina HiSeq2500	Mesp1-induced embryoid bodies	Heterogeneity of the Mesp1^+^ mesoderm cells
Cho et al. [75] PMID: 28076798	January 2017	24	mechanical and enzymatic digestion, FACS	custom plate-based; Illumina NextSeq 500	mESCs-derived CMs and adult CMs (αMHC-GFP)	Comparison mESC-derived CMs differentiation, in vitro or post- implantation
Bektik et al. [62] PMID: 28796841	August 2017	Does not specify	enzymatic digestion from culture, FACS	Fluidigm C1 IFCs; multiplex TaqMan^®^ sc-qPCR	hESC-derived hCMs, hCFs and hiCMs (αMHC-mCherry^+^)	hESC-derived fibroblast differentiation hiCM
Liu et al. [64] PMID: 29072293	October 2017	454	enzymatic digestion, FACS	Fluidigm C1 IFCs; Illumina HiSeq2500	cultured CMs and fibroblasts	fibroblasts to iCM reprogramming
Friedman and Nguyen et al. [68] PMID: 29072293	October 2018	43,168	enzymatic digestion from culture	Chromium Single Cells 3’ v1(10x Genomics); NextSeq 500 (Illumina). Fluidigm C1 IFCs, Illumina’s HiSeq 2000	hiPSC-derived CMs	Multiple time stages of hiPSC differentiation to CM (day 0, 2, 5, 15, 30). Identify HOPX, signal to enhance CM differentiation
Churko et al. [67] PMID: 30464173	November 2018	10,427	enzymatic digestion from culture	Chromium Single Cells 3’ v2 (10x Genomics); NextSeq 500 (Illumina).	hiPSC-derived CMs	hiPSC cardiac differentiation. Multiple time points (day 0, 5, 14, and day 45)
Biendarra-Tiegs et al. [69] PMID : 30892143	April 2019	85	enzymatic digestion	Fluidigm C1 IFCs, Illumina’s HiSeq 2500	hiPSC-derived CM (d12-d40 of differentiation)	hiPSC-derived CM maturation (Electrophysiological vs. transcriptomic profiling)
Zhou et al. [65] PMID: 31230860	June 2019	704	enzymatic digestion, FACS live cells	Fluidigm C1 IFCs, Illumina HiSeq 2500	hCF-induced CMs	Time course of hCF to hiCM reprogramming (d0, d3, d5, d7, d9 post-infection)
Stone et al. [66] PMID: 31271750	July 2019	29,718	enzymatic digestion, FACS	Chromium Single Cells 3’ v2 (10x Genomics); Illumina HiSeq4000	stimulated embryonic CMs and fibroblasts	Time course of mCF to miCM reprogramming (d-1, 0, 1, 7, 14 post-infection)
Raghunathan et al. [79] PMID: 31678351	October 2019	560	enzymatic digestion from culture	Chromium Single Cells 3’ v2 (10x Genomics); NextSeq 500 (Illumina).	Induced cardiac pacemaker-like cells	Human CPs (derived from hASMSC) differentiation to pacemaker-like cells
Ruan et al. [74] PMID: 31722692	November 2019	6879	enzymatic digestion;image-based selection of live cells	Icell8 platform (Takara); Illumina NextSeq500	embryonic cardiac cells	Human ESCs to CM differentiation (d0, 2, 5, 9, 14, and 60)
Gambardella et al. [73] PMID: 31767620	December 2019	362	enzymatic from culture	Smart-seq2; Illumina Nextera XT	hESC-derived epicardial cells	Characterization of epicardial cell heterogeneity
Selewa et al. [70] PMID: 32001747	January 2020	≈50,000	enzymatic digestion from culture, mechanical isolation; Nuclei EZ Prep isolation kit (Sigma)	Drop-seq [12], DroNc-seq [13]; Illumina NextSeq500	hiPSC-derived CM, human cardiac nuclei	ScRNAseq versus snRNAseq on: iPSC to CM differentiation (d0, 1, 3, 7, 15), human heart tissue
Kamdar et al.[71] PMID: 32164890	March 2020	264	enzymatic from culture	does not specify; Illumina MiSeq	hiPSC-derived CMs	CMs derived from control and DMD patients (d30-d60)
He et al. [78] PMID: 32276728	April 2020	11,772	enzymatic digestion from culture	Chromium Single Cells 3’ v2 (10x Genomics); Illumina HiSeq2000	hESC-derived cardiac cells	Effect of nicotine on cardiac differentiation from hESCs
Wang et al. [76] PMID: 32444791	May 2020	2497	enzymatic digestion; image-based selection of live cells	Icell8 platform (Takara); Illumina NextSeq500	murine heart LV; CM-fibroblasts co-cultures	Murine postnatal CM maturation: p1, 4, 7, 14, 56 hearts (LV); in vitro imCM with neonatal or adult fibroblasts
Miao et al. [56] PMID: 32810435	August 2020	32,901 human fetal heart cells (35,284 total)	Dissection, mechanical and enzymatical digestion, MACS	Chromium Single Cells 3’ v2 (10x Genomics); Illumina HiSeq4000	Human fetal heart cell, enrichment for CD144^+^ endo cells; hiPSC-ECs	Hypoplastic left heart syndrome (HLHS): human fetal heart tissue, hiPSC-derived endocardium
Lam et al. [58] PMID: 33059525	October 2020	25,079	Enzymatic digestion	Chromium Single Cells 3’ v2 (10x Genomics); Illumina HiSeq4000	hiPSC-CMs and hiPSC-CMs in anisotropic sheets or cardiac strips	Pulmonary Atresia with Intact Ventricular Septum, a form of HRHS

**Table 3:** Adult Cardiac Progenitor Cells.

Authors PMID	Date	# of Cells and/or Nuclei	Isolation Method	Sequencing Technology	Target Cell Types	Context
Noseda et al. [80] PMID: 25980517	May 2015	128	enzymatic digestion, FACS	Manual RNA extraction; targeted Taqman^®^ sc-qPCR	Adult cardiac progenitor cells	Cardiac cell lineage commitment
Yao et al. [81] PMID: 26043119	June 2015	48	enzymatic digestion, FACS	Fluidigm Dynamic Array IFCs targeted sc-qPCR [6]	Transplanted BM- MSC	Paracrine function of injected cells. Analysis 10 days post-ligation
Ong et al. [82] PMID: 26304668	August 2015	does not specify	Langendorff enzymatic digestion, FACS	Fluidigm Dynamic Array IFCs targeted sc-qPCR [6]	Transplanted hiPSC-CM	Paracrine function of injected cells. Analysis 4 days post-ligation
Chen et al. [77] PMID: 27622691	September 2016	6	enzymatic digestion	Custom microfluidic chip [86]; Targeted sc- qPCR and MG430 2.0 Affimetrix single-cell transcriptome	CMs, CM-derived progenitor cells (mCPCs)	CM de-differentiation
Chen et al. [83] PMID: 29021323	October 2017	405	mechanical and enzymatic digestion, MACS	Fluidigm C1 IFCs; Illumina HiSeq2500	Cardiac CD45−c-kit^+^ cells	Profiling the heterogeneity of c-kit^+^ CPs, from p1 and adult hearts
Kim et al. [84] PMID: 30104715	August 2018	2465 (10x Chromium); 1126 (Smart-seq2)	mechanical and enzymatic digestion, MACS	10x 3’ v2 (10x Genomics), Smart-seq2; Illumina HiSeq2500, NextSeq500.	c-Kit^+^/Lin^−^ CPs freshly isolated and after 5 passages in culture	Comparison of freshly isolated and cultured CPs
Broughton et al. [85] PMID:31231694	June 2019	1664	mechanical and enzymatic digestion, FACS	10x 3’ v2 (10x Genomics); Illumina HiSeq 2500	c-kit^+^ interstitial non-myocytes	Ploidy in cardiac c-kit+ interstitial non-CMs

## 4. Profiling Injury Models in Regenerative Heart

The main animal models used to study cardiac regeneration are neonatal mice, within the first week of age [87], and more ancient vertebrates such as zebrafish, which can undergo cardiac regeneration throughout life [88,89] (Table 4). The first pioneering study, presenting single-cell transcriptomic analysis in zebrafish, used the Fluidigm C1 platform to profile about 30 genetically labeled epicardial cells. The epicardial layer had been previously reported to have an essential role in zebrafish heart regeneration. This study highlighted the heterogeneity within this population and revealed a new pan-epicardial marker important for regeneration and injury-induced CM proliferation [90]. Later, CMs from regenerating zebrafish hearts were profiled by SORT-Seq, 7 days post-cryoinjury [91]. The authors observed that CMs in the border zone had a different profile than those in the remote area, resembling embryonic cells, and they presented a metabolic switch to glycolysis promoting proliferation, which was induced by the ErbB2 signaling. Recently, a larger scRNAseq analysis of all ventricular cells has been used to understand the role of *Runx1* loss of function mutation in zebrafish cardiac regeneration three days post-cryoinjury [92]. *Runx1* appeared to affect the function of different cell types, and the mutant showed less myofibroblast-like cells, less collagen deposition, increased fibrinolysis, and overall enhanced regeneration.

As for cardiac regeneration in neonatal mice, the same group has profiled both CM nuclei [93] and interstitial cells [94], in regenerative (p1) and non-regenerative (p7) mice, sham-operated or 1, 3 days post-MI. The first study identified two factors expressed by proliferating CM in regenerative hearts that could confer protection if overexpressed in non-regenerative adult murine hearts. The second provided both transcriptomic and chromatin-accessibility profiles of all the interstitial cells involved in the regeneration, identifying pro-regenerative factors secreted by the epicardium and by macrophages for the reconstruction of cell-specific gene regulatory networks. Transcription factors such as YAP [95] and PITX2 [96] regulate regeneration in neonatal hearts, partly by modulating antioxidant scavengers’ gene expression, thus protecting the injured myocardium from ROS. Neonatal hearts from a CM-specific *Pitx2* conditional gene knockout presented persistent large scars two months post-MI and adipose infiltrates derived from non-CM cells [97]. Three weeks post-MI, snRNAseq showed a relative increase in a subset of CMs expressing genes associated with oxidative stress response, confirming elevated oxidative stress in *Pitx2*-deficient hearts [97]. Finally, a separate group used scRNAseq to specifically profile CD45^+^CD3^+^ T cell response in regenerative mice [98,99]. In the first study [98], the authors compared naïve T cells isolated from the spleen with activated T cells isolated from the hearts of p3 mice 7 days post-cryoinjury and reported reduced proliferation and an increase in chemotactic factors that could recruit innate immune cells. In the second study, they used the same injury model to compare activated T cells in regenerative p3 hearts (versus non-regenerative p8 heart) and observed elevated fibrotic CD4^+^ T cells and more Th1 Th17 cells in p8 hearts [99]. By CD4^+^ cell depletion, they were able to restore regeneration in juvenile p8 mice, but not in adult mice, suggesting the acquisition of a distinct loss of function with development.

In summary, scRNAseq has been used to identify cell-specific transcriptomic differences in regenerative versus non-regenerative hearts. This analysis has highlighted cellular functions that are specific to cardiac regeneration, which present notable future targets for pharmacologic efforts to improve cardiac repair.

**Table 4:** Cardiac Injury in Regenerative Models.

Authors PMID	Date	# of Cells and/or Nuclei	Isolation Method	Sequencing Technology	Target Cell Types	Context
Cao et al. [90] PMID: 26657776	December 2015	31	enzymatic digestion, FACS	Fluidigm C1 platform, Illumina HiSeq 2000	tcf21^-^ nucEGFP^+^ epicardial cells	Zebrafish cardiac regeneration
Li and Tao et al. [97] PMID: 30143541	September 2018	7849	mechanical nuclei isolation	Chromium Single Cells 3’ v2 (10x Genomics); Illumina Nextseq 500	adult cardiac nuclei	Analysis of *Pitx2* conditional-KO with P2-MI 60 days post-sham or injury
Li et al. [98] PMID: 31285764	June 2019	581 CD3+ heart T cells (1850 from spleen)	mechanical and enzymatic digestion, FACS	Chromium Single Cells 3’ v2 (10x Genomics); Illumina HiSeq 2500	CD3^+^ T-cells	Comparison of naïve T-cells (liver) and Treg (heart) d7 after cryoinjury in P3 mice hearts
Honkoop and de Bakker et al. [91] PMID: 31868166	December 2019	768	mechanical and enzymatic digestion	SORT-seq [9], plate-based	embryonic, adult zebrafish cardiac cells	Comparison of embryonic (2dpf) and regenerating CMs (7d cryoinjury)
Cui et al. [93] PMID: 32220304	March 2020	21,737	mechanical and enzymatic digestion, nuclei isolation, FACS	Chromium Single Cells 3’ v2 (10x Genomics); Illumina Nextseq 500	CM nuclei	Neonatal and postnatal regenerative capacity: CM from P1 or P8 mice sham, d1, d3 post-MI
Koth et al. [92] PMID: 32341028	April 2020	15,415	mechanical and enzymatic digestion, FACS	Chromium Single Cells 3’ v2 (10x Genomics); Illumina HiSeq 4000	adult zebrafish cardiac cells	Runx1 KO zebrafish cardiac regeneration
Li et al. [99] PMID: 32724455	June 2020	2431	mechanical and enzymatic digestion, FACS	Chromium Single Cells 3’ v2 (10x Genomics); Illumina HiSeq 2500	cardiac and splenic T-cells	Neonatal cardiac regeneration after apical resection and cryoinfraction
Wang et al. [94] PMID: 33296652	December 2020	17,320	mechanical and enzymatic digestion	Chromium Single Cells 3’ v2 (10x Genomics); Illumina Nextseq 500	Interstitial cells	Neonatal and postnatal regenerative capacity: interstitial cells from P1 or P8 mice sham, d1, d3 post-MI

## 5. Profiling Cardiac Diseases and Injury Models in Non-Regenerative Hearts

One of the classical applications of transcriptomic analysis is the comparison of healthy versus diseased tissues. Single-cell analysis allows for the characterization of transcriptomic changes during disease within individual cells, but also within entire cell populations that comprise a tissue (Table 5).

Initial cardiac scRNAseq studies on disease models were mainly focused on a specific selected/enriched population of cells. The first attempt to profile the CM response in failing hearts was done on a model of pressure-overload induced through TAC, using the Fluidigm C1 platform [100]. Nuclei were isolated from murine hearts, 8 weeks post-TAC, and from biopsies of patients at the late stage of DCM [100]. Despite the limited number of sequenced cells, the depth of paired-end sequencing and the single-cell resolution led to the identification of two long non-coding intragenic RNAs (lincRNA) that could regulate CM cell cycle re-entry and de-differentiation [100]. A comparable number of full CMs was sequenced, after manual isolation and lysis, with the SmartSeq2 protocol, from sham- and TAC-operated mouse hearts at multiple time points post-surgery (3 days, 1, 2, 4, 8 weeks) [101]. This study identified activated pathways and reconstituted the transcriptional trajectory of remodeling CMs, which showed two distinct fates: adaptation and failing. The same group has later combined scRNAseq by SmartSeq2, with sc-qPCR and smFISH, to obtain spatial information on the heterogenous CM response to pressure overload. They observed that the middle myocardium layer was more affected, with re-expression of the fetal gene *Myh7* [102]. More recently, they have used a similar approach to sequence additional CM from sham and TAC-operated mice, and human patients with severe heart failure (HF) [103], and they have showed that the dopamine receptor 1 is the only catecholamine receptor significantly upregulated in failing hearts and contributes to the ventricular arrhythmia observed in chronic HF patients treated with dopamine. A separate group used an image-based platform to isolate, sequence, and compare mono- and pluri-nucleated CM in homeostasis and 8 weeks post-TAC [104], and they concluded that the differences in ploidy do not correspond to significant transcriptional differences in homeostasis, as well as that the heterogeneity observed post-injury is mainly attributable to the non-homogeneous oxygen distribution.

Two studies have used the SORT-Seq method [9] to profile both CM and non-CM after FACS, sorting them in 384-well plates [105,106]. The first study [105] analyzed the infarct area and border zone region, 3 days post-ischemia/reperfusion (I/R), and the correspondent LV area in control mice, reporting *Cfk4* as a new marker of activated fibroblasts. The second study [106] combined scRNAseq of about 2000 cells, and lineage tracing using a Ki67 knock-in reporter mouse, to investigate proliferating cells in different conditions: adult and neonatal hearts, sham, I/R or MI hearts (scar and distal area) 3-, 7-, and 14-days post-ligation. They found no evidence of quiescent cardiac stem cells or proliferating CM in the adult heart, but the result could be affected by the limited number of cells sampled in each condition. They observed that proliferating adult fibroblasts prevent cardiac rupture post-damage and acquire a neonatal phenotype post-injury with the expression of follistatin-like protein 1 (*Flst1)*.

Given their large size and fragile structure, the isolation of CMs by FACS could lead to the capture of damaged cells or cell fragments, especially if the cell sorting is only aimed to remove DAPI^+^ dead cells [107]. As we describe below, manual or automatic cell dispersion combined with imaging selection, as well as single nuclei isolation, are still considered the most reliable ways to isolate CM for sequencing. The first approach allows for the acquisition of morphological and phenotypic information on the sequenced CMs, including the ploidy level; the second can be applied both on live and frozen tissues, and it is advantageous for a high-throughput sampling of a large number of cells.

The image-based system ICell8 CellSelect (Takara) has been used in two studies [108,109] to automatically select single live nucleated CM and non-CM distributed in nanowells. The first study [108] focused on analyzing CM and non-CM from the atria and ventricle of human healthy, failing, and partially recovering heart post-LVAD (left ventricular assist device) treatment, providing a resource to investigate inter- and intra- compartmental heterogeneity in response to stress. The second [109] analyzed the progression toward HF, profiling hearts of mice exposed to TAC at different stages (0, 2, 5, 8, 11 weeks), and showed changes in cell–cell communications, subtype switching in fibroblasts, and activation of pro-inflammatory macrophages in the mid-stage of HF progression that can be targeted to preserve cardiac function. Similar transcriptomic changes were observed in human samples of HF and hypertrophy.

Other studies have used snRNAseq to sample CMs, and they either used total snRNAseq [110] or a combination of snRNAseq and scRNAseq [111] to sequence interstitial cells and CMs in pathological contexts. The first study [110] sequenced nuclei from all cardiac cell types isolated from sham or MI murine hearts 5 days post-surgery. The authors used a tri-transgenic mouse line to differentiate between pre-existing, de novo differentiated, and de-differentiated CM. They observed no *de novo* CM differentiation from interstitial cells, but a small population of differentiated CM appeared to re-enter the cell cycle. The second study [111] analyzed CM nuclei and interstitial cells in a murine model of hypertension, which was induced with 2-weeks AngII infusion, comparing control and treated mice of both sexes. The analysis revealed the transcriptomic signature of activated fibroblasts responsible for the perivascular and interstitial fibrosis as well as gene expression differences based on biological sex evident in almost all cell types, particularly in fibroblasts. A recent study [112] has used published snRNAseq [100] and scRNAseq [101] on mouse CMs post-TAC and human hearts with DCM [101], with newly generated scRNAseq data of mouse interstitial cells 7 days post-MI, to prioritize possible biomarkers identified through patient plasma proteomic analysis.

Many studies have selectively focused on the non-CM component of the heart using the high throughput 10x Chromium technology. One early study [113] profiled both primary fibroblasts response to TGFß and the interstitial cells of a genetic mouse model of fibrosis (carrying a mutation in phospholamban PLN^R9C/+^), showing that Il11 is a primary downstream effector of TGFß in fibroblasts: its overexpression causes heart and kidney fibrosis, the downregulation protects from fibrosis and organ failure. Two additional studies profiled the interstitial cell response to MI in an unbiased fashion. One analyzed the response of the main cell populations, 3- and 7-days post-ligation, using *Pdgfra-GFP* mice for the lineage tracing of potential progenitor-like cells [114]. The second profiled seven-time points across the three main phases of cardiac repair: homeostasis (d0), inflammatory (d1), proliferative (d3-d5-d7), and maturation (d14-d28) phase, using a reporter mouse to discriminate epicardial from endocardial-derived fibroblasts (*Wt1Cre* x *ZsGreen* mice); and they focused on the different fibroblast types/states prevalent in each phase [115]. Additionally, the authors compared genetically diverse inbred mouse strains characterized by different reparative outcomes to highlight the cell composition and transcriptomic features associated with a higher frequency of cardiac rupture in the transition between the inflammatory and proliferative phases. Three studies selectively profiled the fibroblast component of the heart [116,117,118]. The first early study [116] used the Fluidigm C1 platform to profile several hundred CD31-CD45 cells isolated from the scar area of inducible reporter mice for *Tcf21* or *Postn*, 7 days post-MI. Based on the expression of known markers, the authors confirmed that *Postn*-traced cells accounted for most of the myofibroblasts in the scar and were derived from *Tcf21*^+^ fibroblasts. The other two studies adopted the high-throughput 10x Chromium technology. One study [117] analyzed cells labeled for *Pdgfra* and *Hic1* expression, in homeostasis and 7 days post-MI, and it showed that the quiescence gene *Hic1* is expressed by *Sca1*^+^ *Pdgfra*^+^ cells, which act as progenitors for *Sca1* fibrogenic cells post-injury and contribute to pathogenesis rather than regeneration, in contrast to the equivalent cells in skeletal muscle [119]. The second study [118] analyzed *Col1a1-GFP^+^* fibroblasts in homeostasis and 7, 14, and 30 days post-MI. The authors identified *Cd200* as a marker of infarct repair fibroblasts and proved that *Cthrc1* expression (previously reported as a marker of myofibroblasts/activated fibroblasts) is essential, since the KO increases the frequency of cardiac rupture, which is a finding confirmed by bulk RNAseq on swine hearts and patients with MI and DCM. The response of endothelial cells to MI was analyzed in detail, combining clonal lineage tracing with an inducible reporter mouse (*Pdgfb-iCreER^T2^-R26R-Brainbow2.1)* and scRNAseq on sorted ECs, in homeostasis and 7 days post-ligation [120]. As a result, resident ECs appeared to be the main contributors to new vessels through clonal expansion, and *Plvap* was identified as a marker of activated/proliferating ECs, which is also expressed in human samples, and it is potentially targetable to improve neovascularization. A recent study has shown that the expression of VEGF-B in CM can stimulate neoangiogenesis and limit the cardiac damage post-MI in adult murine heart [121]. Combining lineage tracing and scRNAseq data, the authors observed that the new vessels were mostly derived from sub-endocardial ECs [121].

Leukocytes have been selectively enriched and profiled in response to MI [122] in a model of pressure–volume overload [123] and experimental myocarditis [124]. The comparison of leukocytes from infarcted and non-infarcted murine hearts 4 days post-surgery has contributed to identifying a subset of interferon-inducible macrophages and an IRF3–interferon axis that could be targeted to reduce inflammation and improve cardiac function [122]. More recently, the same authors have analyzed myeloid cells in the serum of human patients 28 h post-NSTEMI and in mice serum and hearts 1, 2, and 4 days post-MI [125]. They have shown that the expression of interferon-stimulated genes (ISG) starts in the bone marrow and in circulating neutrophils and monocytes (controlled by the transcription factors *Tet2* and *Irf3*), while within the heart, *Nrf2* negatively regulates ISG expression in resident *Ccr2*^-^ macrophages. They proposed the use of the ISG score, from blood single-cell analysis, as a prognostic tool to stratify patients who may benefit from anti-inflammatory therapies. CD45^+^ leukocytes have also been profiled in a model of pressure-overload induced through TAC, where differences between control hearts and early and late stages of remodeling (1–4 weeks post-surgery) revealed dynamic changes in cells from both the innate and adaptive immune system [123]. Similarly, CD45^+^ cells were isolated and sequenced from murine hearts exposed to an experimental autoimmune model (EAM) at different stages: acute, subacute, myopathy phases, and controls [124]. The authors observed a prevalence of macrophages in every stage; neutrophils appeared in the early stage, and T cells mostly appeared in the subacute phase. Both pro-inflammatory macrophages and Th17 cells showed an upregulation of *Hif1a*, which correlated with the extent of inflammation. *Hif1a* inhibitor could reduce inflammation in all the stages of EAM, and it could be potentially targeted for treatment, as it was expressed at a higher level in patients with acute myocarditis compared to DCM and controls.

The myeloid cell response to MI has been characterized in depth by first profiling sorted resident macrophages and dendritic cells and then sequencing randomly isolated mononuclear cells from adult hearts control at 2, 11, and 28 days post-MI [126]. This study showed that resident embryonic-derived CCR2^-^ macrophages have a non-redundant cardioprotective function that cannot be compensated by the highly similar monocyte-derived TIMD4^-^CCR2^-^ macrophages post-MI. In human, peripheral CD31^+^ monocytes were profiled in patients with HF with reduced ejection fraction (HFrEF) and healthy individuals, revealing profound phenotypic differences, which were validated with different techniques that could be used as a prognostic tool [127].

Recent advances in single-cell technologies are presenting new opportunities to capture increasing amounts and types of information simultaneously from the same cell. For example, cellular indexing of transcriptomes and epitopes by sequencing (CITEseq) quantifies the expression of surface antigen using DNA-barcoded antibodies, while paired full-length sequencing of T or B cell receptors reveals the specific clonotype of adaptive immune cells.

CITEseq has been used to profile the dynamic changes of neutrophils in response to MI. CD11b^+^ live cells were sorted from murine hearts and blood 1, 3, and 5 days post-MI. DNA barcoded antibodies for LY6G, CD64, and LY6C were used to distinguish between neutrophils and monocytes/macrophages [128]. Heart-infiltrating neutrophils appeared to acquire a distinct signature (SiglecF^hi^), while circulating ones underwent aging by d3-d5.

Paired single-cell TCR- and 5′ gene expression sequencing has been recently applied to profile the clonotype of CD4^+^ T cells isolated from the heart and spleen of mice 7 days post-MI. Interestingly, the authors observed that Treg cells are mostly recruited to the heart from the circulating pool, and that they acquire unique features in the tissue, proliferate locally by clonal expansion, and contribute to collagen deposition and repair [129].

Overall, single-cell transcriptomic approaches have been widely employed to study cardiovascular disease, either with a targeted approach, to profile cell-specific changes in response to injury or in an unbiased fashion. The latter studies have been used to address a wide variety of questions, including defining organ-wide changes in cell composition and cell–cell communications, as well as pinpointing how these processes may differ based on biological sex or genetic background. Together, these data provide valuable insights on possible therapeutic targets. As the technology becomes more accessible, compositional shifts and transcriptomic patterns associated with disease could also be used for diagnostic and prognostic screenings.

**Table 5:** Cardiac Injury in Non-Regenerative Models.

Authors PMID	Date	# of Cells and/or Nuclei	Isolation Method	Sequencing Technology	Target Cell Types	Context
Kanisicak et al. [116] PMID: 27447449	July 2016	185	mechanical and enzymatic digestion, FACS	Fluidigm C1 IFCs; Illumina HiSeq2500	CD31-CD45- cardiac cells	Tcf21 lineage tracing during adult MI, TAC and/or AngII infusion
See et al. [100] PMID: 28790305	August 2017	359	mechanical nuclei isolation	Fluidigm C1 IFCs; Illumina HiSeq2500	Adult human and murine CMs	CM response to heart failure: Human DCM, mouse TAC (8 weeks)
King et al. [122] PMID: 29106401	November 2017	4215	enzymatic digestion	InDrop [14]; Illumina HiSeq2500	leucocytes	IFNr in leucocytes, CTR and d4 post-MI
Schafer et al. [113] PMID: 29160304	November 2017	4548	enzymatic digestion	Chromium Single Cells 3’ v2 (10x Genomics); NextSeq 500 (Illumina)	adult cardiac non-myocyte	wt versus Pln^R9C/+^ mouse (cardiac fibrosis phenotype). Il11 mediator of fibroblast activation via TGFb
Gladka, M.M. et al. [105] PMID: 29386203	January 2018	932	enzymatic digestion, FACS (DAPI, scatter properties)	plate-based, SORT-seq; Illumina NextSeq	adult CMs, endothelial cells, fibroblasts, and macrophages	Uninjured LV versus ischemic area 3d post-IR. Cfk4 regulator of fibroblast activation post-injury
Nomura et al. [101] PMID: 30375404	October 2018	482	Langendorff perfusion, manual pipette	Manual CM lysis, cDNASmart-seq2; Illumina HiSeq 2500	adult murine CMs	CMs response to pressure-overload. Sham, 3d and 1, 2, 4, 8 wks post-TAC
Kretzschmar and Post et al. [106] PMID: 30530645	December 2018	1939	Mechanical and enzymatic digestion,FACS (DAPI, MitoTracker)	CEL-Seq2 and TruSeq library preparation for NextSeq500	All murine adult ventricular cells	Ki67-RFP mouse model to assay proliferation during murine cardiac injury
Dick et al. [126] PMID: 30538339	December 2018	8283	enzymatic digestion, Ig based FACS on beads enriched CD45^+^ population	Chromium Single Cells 3’ v2 (10x Genomics); Illumina HiSeq2500	adult mononuclear phagocytes (CD45^+^ CD64^Dim–Hi^ CD11b^+^)	Profiling macrophages post-murine MI (CTR, d2, d11, d28)
Satoh et al. [102] PMID: 30611794	January 2019	219	Langendorff perfusion, manual pipette	Manual cell picking, SMART-seq2, HiSeq 2500 System	adult CMs	Spatial and temporal CMs response to pressure-overload. (sham, 1, 2, 8 wks post-TAC)
Farbehi et al. [114] PMID: 30912746	March 2019	30,118	enzymatic digestion, FACS	Chromium Single Cells 3’ v2 (10x Genomics); Fluidigm C1 IFCs, Illumina’s HiSeq 2500	TIP cells and adult cardiac non-myocytes	Murine MI (Sham, d3, d7)
Zhang et al. [110] PMID: 31231540	June 2019	31,542	mechanical isolation and lysis from fresh frozen tissue	10x Chromium Single Cell 5’ kit (10x Genomics); Illumina HiSeq2500	adult cardiac nuclei	Murine MI (control and d5), tri-transgenic mouse line for CM lineage tracing
Li et al. [120] PMID: 31162546	August 2019	≈28,000	enzymatic digestion, FACS	Chromium Single Cells 3’ v2 (10x Genomics); Illumina HiSeq4000	adult cardiac endothelial cells	Murine MI (control and d7), reporter mouse for clonogenic tracing of ECs
Yekelchyk et al. [104] PMID: 31399804	August 2019	1,301	Langendorff enzymatic perfusion	ICell8 platform (Takara); Illumina Nextera XT	adult CMs mono- and multi-nucleated	CM profiling in CTR hearts and 8-weeks post-TAC
Martini et al. [123] PMID: 31661975	October 2019	17,853	mechanical and enzymatic digestion, FACS	Chromium Single Cells 3’ v2 (10x Genomics); Illumina NextSeq500	Adult cardiac leukocytes	Murine pressure-overload model (1- and 4-weeks post-sham, TAC operation)
Wang et al. [108] PMID: 31915373	January 2020	21,422	mechanical and enzymatic digestion, image based live cell selection in 384w plates	ICell8 CellSelect (Takara), plate-based lysis and cDNA synthesis SMARTScribe; Illumina NextSeq500	adult human CMs and non-CMs from LA/ LV, RV	Human heart failure: healthy donors, HF caused by coronary disease, partial recovery (LVAD treatment)
Soliman et al. [117] PMID: 31978365	February 2020	32,313	enzymatic digestion, FACS	10x 3’ v2 (10x Genomics), Illumina NextSeq500	Pdgfra-eGFP/ Hic1+ cells in homeostasis; Pdgfra-eGFP cells post-MI	Cardiac stromal progenitor response to injury (apical area d0, d7, d14, d28 post-MI)
Ren et al. [109] PMID: 32098504	February 2020	11,492	mechanical and enzymatic digestion	ICell8 CellSelect (Takara), MSND Wafergen	Murine and human heart CMs and non-CMs	Murine pressure-overload model (0, 2, 5, 8, 11 weeks); human heart failure
Forte et al. [115] PMID: 32130914	March 2020	36,847	mechanical and enzymatic digestion, FACS live cells [130]	Chromium Single Cells 3’ v2 (10x Genomics); Illumina HiSeq Xten	Adult murine heart non-myocytes	Murine MI (d0, d1, d3, d5, d7, d14, d28), epicardial lineage tracing, and mouse diversity
Abplanalp et al. [127] PMID: 32311026	April 2020	181,712	MACS magnetic sorting	Chromium Single Cells 3’ v2 (10x Genomics); Illumina HiSeq4000	Human circulating monocytes (CD31^+^)	Effect of heart failure on circulating monocytes: Healthy versus HFrEF patients
Hua et al. [124] PMID: 32431172	May 2020	34,665	mechanical and enzymatic digestion, FACS live cells	Chromium Single Cells 5’ v2 (10x Genomics); Illumina HiSeq4000	CD45^+^ immune cells	Experimental autoimmune myocarditis: d0, d14, d21, d60 post-induction in Balb/c mice
McLellan et al. [111] PMID: 32795101	July 2020	29,558	perfusion-based enzymatic digestion, FACS	Chromium Single Cells 3’ v2 (10x Genomics); Illumina HiSeq4000	adult cardiac non-myocyte cells and CM nuclei	Murine hypertension (Sham, AngII- 2wks post-treatment), male and female comparison
Vafadarnejad et al. [128] PMID: 32811295	August 2020	1334	mechanical and enzymatic digestion, FACS (CD11b^+^ live cells)	CITE-seq, Chromium Single Cells 3’ v2 and v3 (10x Genomics); Illumina NovaSeq6000	adult cardiac neutrophils	Neutrophils dynamics post-murine MI (d1, d3, d5)
Yamaguchi et al. [103] PMID: 32868781	August 2020	280 murine CMs and 514 human CMs	Langendorff enzymatic perfusion, manual pipette	Smart-seq2, Illumina HiSeq2500	Adult murine and human CMs	Interrogation of cardiac dopamine receptor expression during arrhythmia in mice and heart failure in humans
Chan et al. [112] PMID: 32885678	September 2020	830 mouse non-myocyte cells (additionally utilized publicly available data [100,101])	mechanical and perfusion-based enzymatic digestion, FACS	SMART-Seq2, Illumina HiSeq2000	LV interstitial cells 7 days post-MI, CM nuclei 8 weeks post-TAC	Identify HF biomarkers combining plasma proteomic analysis and scRNAseq
Ruiz-Villalba et al. [118] P MID: 32972203	September 2020	36,858	mechanical and enzymatic digestion, FACS (Col1a1-GFP, CD31, CD45)	10x Genomics 3’ v2; Illumina NextSeq500	Adult cardiac fibroblasts, endothelial, immune cells	Murine MI (d0, d7, d30), Cthrc1-KO MI (d7), swine MI (d7)
Calcagno and Ng et al. [125] PMID: 32978242	Sept 2020	10,666 murine hearts (~145,000 total)	mechanical and enzymatic digestion, FACS (DAPI-, Ter119−)	inDrop [14] and 10x Genomics; Illumina HiSeq2500 and HiSeq4000	Myeloid cells (neutrophils, monocytes, resident macrophages)	IFNr in leucocytes: human serum 28h post-NSTEMI, mouse heart d1, d2, d4 post-MI
Xia and Lu et al. [129] PMID: 32985264	September 2020	20,755 heart T-cells (and 23,741 spleen T-cells)	mechanical and enzymatic digestion, FACS live cells	Chromium Single Cells 3’ v2 and v3 (10x Genomics); Illumina NovaSeq6000	murine cardiac and splenic CD4^+^ T cell TCR sequencing	Profiling Treg in the heart, after MI, I/R, cryoinjury
Räsänen et al. [121] PMID: 33203221	November 2020	does not specify	mechanical and enzymatic digestion, FACS	Chromium Single Cells 3’ v3 (10x Genomics); Illumina NovaSeq6000	cardiac endothelial cells (CD31^+^ CD45^-^ Ter119^-^ CD140a^-^ DAPI^-^)	ECs from CTR and —V-–VEGF-B transduced adult hearts

## 6. Cardiac scRNAseq Cell Atlases

Single-cell transcriptomic analysis deepens our understanding of tissue complexity as well as the dynamic nature of tissue composition and provides a baseline reference for comparison with diseased states. Numerous healthy single-cell atlases have already been developed using the mouse as a model system, although their scope ranges from studies of a specific cell type (e.g., all endothelial cells, from multiple organs) or organ (e.g., all cells present in the heart), to sampling of almost every tissue type within an organism (Table 6).

In 2018, two groups published the first multi-organ compendia of mouse single-cell data, together comprising 500,000 cells from over 50 different organ or tissue types [10,131]. The first, the Mouse Cell Atlas (MCA) [10], simultaneously described Microwell-Seq, which is a novel scRNAseq method utilizing well-based single-cell capture in agarose. The initial MCA contained ≈5000 neonatal heart cells which made up <5% of the total dataset. The website developed in conjunction with this paper has subsequently been updated to MCA 2.0 with additional scRNAseq data from fetal to aged (24 months of age) murine cardiac tissue, totaling over 60,000 heart cells (>800,000 total cells), providing a valuable resource to investigate cardiovascular aging (http://bis.zju.edu.cn/MCA/index.html (accessed on 17 February 2021)). In the second study, the *Tabula Muris* [131], the authors profiled 100,000 cells from 20 murine tissues using two methods: FACS sorting in plates, combined with Smart-seq2 [8] for sequencing of full transcripts; and microfluidic droplet-based cell isolation, for higher throughput 3′-end short-read sequencing. About 4000 cardiac cells were analyzed with the first method and a few hundred were analyzed with the second one (https://tabula-muris.ds.czbiohub.org/ (accessed on 17 February 2021)). Later, the same consortium used a similar approach to profile 23 organs in male and female mice at six age points with the droplet-based system and three time points using the FACS-based method, ranging from 1 to 30 months [132]. This large dataset, with over 350,000 cells, constitutes the *Tabula Muris Senis* and includes about 18,282 cardiac cells [132,133]. Transcriptomic changes occurring with age have been also specifically analyzed in the heart, using murine [134] and non-human primate [135] models. The first study [134] profiled 12-week-old and 18-month-old C57BL/6JRj mice, revealing significant changes in fibroblasts, with upregulation of pro-inflammatory, anti-angiogenic, and osteogenic genes. The second [135] utilized scRNAseq to produce a single-cell compendium of cardiovascular aging in cynomolgus monkeys and reported an increase in inflammatory genes both in immune and non-immune cardiac cells.

The broad scope of studies such as MCA and *Tabula Muris* enable the interrogation of cell identity and tissue-specific aspects of common cell types such as endothelial cells, fibroblasts, and immune cells. An alternative approach to large, multi-organ total cell atlas studies is to narrow the scope of the investigation to a specific cell type. The murine endothelial cell atlas [136,137] consists of more than 32,000 endothelial cells from 11 different mouse tissues. The analysis of this dataset revealed transcriptomic similarity between tissue pairs, and the marker genes of common, tissue-specific, and new EC subtypes (i.e., IFN-responsive and angiogenic ECs). Cardiac endothelial cells appeared transcriptionally similar to skeletal muscle endothelial cells and enriched for genes involved in membrane transport and redox homeostasis. A database of fibroblasts and vascular mural cells has been obtained by sequencing cells isolated from four muscular tissues, including the heart [138,139]. This study revealed lists of genes that can be used to discriminate between these two mesenchyme-derived cell types. Furthermore, the authors observed a higher degree of heterogeneity among fibroblasts from different organs, compared to mural cells, which was mostly ascribable to differences in the ECM genes and that fibroblast subtypes tend to localize in distinct anatomical positions. In the heart, similarly to other studies [10,113,135], they identified two main fibroblast sub-populations. The smaller sub-population, expressing relatively higher levels of *Wif1* and *Dkk3*, was transcriptionally similar to fibroblasts located in the perimysium, which is the connective tissue surrounding a group of myofibers in the skeletal muscle. In the cardiac context, these cells were mostly localized in the valve and atrioventricular space [138], and they seem to resemble interstitial valve fibroblasts [140] and endocardial derived fibroblasts [115] described in separate studies. B cells have been profiled in adult heart [141] and compared across multiple tissues [142] (heart, liver, lung, and blood) in order to identify specific gene expression patterns. These data revealed that the naïve organ-associated B-cells share features that distinguish them from circulating B cells, but they also display tissue-specific gene expression patterns of unclear function [142]. Transcriptional changes in cardiac B cells during development reflect an unexpected constant dynamic equilibrium within the B cells from primary lymphoid organs, such as the spleen, even in the absence of injuries [142]. Single-cell analyses such as these facilitate the development of cell-specific atlases and provide insight into cellular identity and organ-specific functions of common cell types.

An alternative to cell type-specific scRNAseq (i.e., all B cells) is an organ-specific approach. One of the first cardiac-specific single-cell atlases reported was by Skelly et al. in 2018 [143]. This study identified cell type diversity within cardiac ventricular tissue from C57BL/6J mice, tested intercellular paracrine support between cardiac fibroblasts and macrophages, and described distinct gene expression profiles between male and female cardiac cells [143]. The study was limited by the cell isolation method, which required an artificial down-sampling of the endothelial cells and excluded CMs and atria. A later series of studies [144,145,146] used snRNAseq to unbiasedly profile the heart of outbred *Fzt:DU* mice [144] and compare it to inbred C57BL/6NRj mice heart [145] as well as to previously published *Tabula Muris* cardiac data [146]. The authors noted significant differences in cardiac tissue composition between strains, namely twice as many total endothelial cells in Fzt:DU cardiac tissue [145], a cluster of “endothelial like CMs” [144], and a small population of possibly cycling CMs [146]; these interesting observations are still awaiting validation and further interrogation.

Additional cardiac scRNAseq studies further narrow the scope to focus on the analysis of organ sub-structures [147] or specific cell-types during uncharacterized processes such as CMs in culture [148,149]. A detailed analysis of the murine sinus node has been recently obtained by combining quantitative proteomic data of nodal and non-nodal atrial tissue, with snRNAseq transcriptomic data [147]. Using this approach, the authors identified enriched ion channel proteins and assigned them to specific cell types, thus shedding light on the molecular basis of the pacemaking activity in nodal CMs. Single-cell analysis of CMs is also useful to study CM behavior in culture. One study profiled the transcriptomic response of primary rat cardiac cells adapting to a 3D culture environment [148], while another analyzed the effect of the structural geometry present during culture on primary neonatal rat CMs gene expression [149]. The authors noted that CMs that were forced to adopt a square shape (as opposed to the endogenous rectangle shape) upregulated markers of cell death and downregulated essential cardiac signaling pathways such as oxidative phosphorylation. Regardless of scope, single-cell atlases are a valuable resource for murine cardiac biology.

Human cardiovascular biology is also aided by the development of healthy single-cell atlases. One of the first reported human heart single-cell atlases included >280,000 nuclei from all four heart chambers from male and female donors [150]. The authors showed significant (and expected) transcriptional differences between atrial and ventricular CMs, as well as a surprising amount of variation in non-myocyte gene expression based on anatomical location such as a unique atrial fibroblast subtype. Additionally, the authors integrated snRNAseq data with Genome-Wide Association Studies (GWAS) cardiometabolic traits and the Druggable Genome [151] to link disease-relevant SNPs and druggable gene targets to specific cell types [152]. The data can be explored from the Broad Institute Single Cell Portal [153]. A more recent human cardiac single-cell atlas comprised nearly 500,000 cells and nuclei from the four chamber walls, as well as the septum and apex [154]. The authors carefully defined the heterogeneous composition of all the main cardiac cell populations and validated the spatial distribution of selected clusters by smFISH. Additionally, they identified the cell types enriched for genes associated with cardiovascular phenotypes and SARS-CoV-2 infection (atrial fibrillation, PR interval, QRS duration, coronary artery disease, and hypertension diseases) from 12 GWAS studies [155]. The data can be explored at [156].

In summary, scRNAseq of the uninjured heart is a useful tool to better define cardiac cellular identity and tissue composition. Single-cell transcriptomic analysis enables the generation of high-resolution cell atlases to delineate a map of the heart, providing insights into the composition of a healthy heart so as to detect deviations into pathogenesis.

**Table 6:** Adult heart single-cell atlases.

Authors PMID	Date	# of Cells and/or Nuclei	Isolation Method	Sequencing Technology	Target Cell Types	Context
Skelly et al. [143] PMID: 29346760	January 2018	10,519	enzymatic digestion, FACS	Chromium Single Cells 3’ v2 (10x Genomics); Illumina HiSeq 4000	adult cardiac non-myocyte	homeostatic murine adult cardiac tissue. Male and female comparison
Han et al. [10] PMID: 29474909	February 2018	5075 heart cells (Over 400,000 total)	enzymatic digestion	Microwell-Seq; Illumina HiSeq	neonatal cardiac cells	Mouse Cell Atlas
Tabula Muris Consortium [131] PMID:30283141	October 2018	4635 heart cells (over 100,000 total)	enzymatic digestion, FACS, manual pipette	GemCode Single-Cell 3’ v2 (10x Genomics) & FACS-based full length transcriptomic; Illumina NovaSeq 6000	adult cardiac non-myocytes, CM	Homeostatic cell profiling of 20 murine adult organs
Hulin et al. [140] PMID: 30796046	March 2019	2840	mechanical and enzymatic digestion	Dropseq	heart valve cells	Aortic valve and mitral valve at P7 and P30
Linscheid et al. [147] PMID: 31253831	June 2019	5357	Mechanical nuclei isolation	Chromium Single Cells 3’ v3 (10x Genomics); Illumina NovaSeq 6000	sinus node nuclei	Murine sinus node cell atlas
Wang et al. [148] PMID: 31455969	August 2019	12,865	mechanical and enzymatic digestion, FACS	10x 3’ v2 (10x Genomics); Illumina HiSeq PE150	3D-cultured primary cells	Engineered cardiac tissues (derived from rat primary cells)
Vidal et al. [134] PMID: 31723062	September 2019	27,808	Mechanical nuclei isolation	Chromium Single Cells 3’ v3 (10x Genomics); Illumina Hiseq4000	adult CM and non-myocyte nuclei	Young and aged C57BL/6JRj mice
Haftbaradaran Esfahani et al. [149] PMID: 31872302	December 2019	435	Enzymatic from culture and semi-automatic cell picking [157]	Smart-Seq2; Illumina HiSeq 2500	cultured primary p2 rat CMs	Profiling of CMs with defined morphotypes through custom geometry culture chips [158]
Adamo et al. [141] PMID: 31945014	January 2020	5571	mechanical and enzymatic digestion, FACS	Chromium Single Cells 3’ v3 and 5’ V(D)J enriched library (10x Genomics); Illumina NovaSeq6000	CD45^+^Aqua–CD19^+^ B-cells	B-cells from the heart and blood of 10-week-old C57BL/6J mice
Wolfien et al. [144] PMID:32013057	January 2020	8635	mechanical and enzymatic digestion, Nuclei PURE Prep isolation kit (Sigma)	Chromium Single Cells 3’ v3 (10x Genomics); Illumina NovaSeq6000	adult CM and non-myocyte nuclei	snRNAseq whole murine heart (Fzt:DU outbred mice)
Kalucka et al. [136] PMID: 32059779	Feb 2020	4612 heart endothelial cells (32,567 total cells)	mechanical and enzymatic digestion, FACS	Chromium Single Cells 3’ v2 (10x Genomics); Illumina Hiseq 4000	Adult murine heart endothelial cells	Murine endothelial cell atlas from 11 tissues
Wolfien, Galow, and Müller et al. [145] PMID: 32243511	April 2020	3464 nuclei (additionally integrated with previously published data [144])	mechanical and enzymatic digestion, Nuclei PURE Prep isolation kit (Sigma)	Chromium Single Cells 3’v3 (10x Genomics); Illumina NovaSeq6000	adult CM and non-myocyte nuclei	snRNAseq whole murine heart (Fzt:DU outbred vs. C57BL/6NRj mice)
Tucker and Chaffin et al., [150] PMID: 32403949	May 2020	287, 269	mechanical and enzymatic digestion, nuclei isolation	Chromium Single Cells 3’ v2 (10x Genomics)	all human heart cell types	Healthy human adult cardiac tissue: biopsies from four chambers
Rocha-Resende et al. [142] PMID: 32663200	July 2020	1004	mechanical and enzymatic digestion, FACS	Chromium 10x 3’ v3 and 5’ (10x Genomics); Illumina NovaSeq 6000	CD45^+^Aqua–CD19^+^ B-cells	B-cells from postnatal (2 wks) and adult hearts (8 wks); comparison with other tissues (10 wks)
Tabula Muris Consortium [132] PMID: 32669714	July 2020	18,282 heart cells over 350,000 total (9,669 cells long-reads; 8,613 short-reads)	enzymatic digestion, FACS, manual pipette	GemCode Single-Cell 3’ v2 (10x Genomics) & FACS-based full length transcriptomic; Illumina NovaSeq 6000	adult cardiac non-myocytes, CM from all four chambers	Profiling of 23 murine adult organs over six age stages (1 to 30 months), male and female C57BL/6J.
Muhl et al. [138] PMID:32769974	August 2020	1,279 heart cells (6158 total)	mechanical and enzymatic digestion, FACS	Smart-Seq2; Illumina HiSeq 3000	PDGFRa^+^, PDGFRb^+^, CD31^-^ ;	Comparison of fibroblasts and mural cells from four different organs
Ma et al. [135] PMID: 32913304	September 2020	42,053 (109,609 additional lung nuclei)	mechanical nuclei isolation and FACS	Chromium Single Cells 3’ v3 (10x Genomics); Illumina NovaSeq6000	young and aged primate cardiac nuclei	Lung and heart from young and aged cynomolgus monkeys
Litviňuková et al. [154] PMID: 32971526	September 2020	487,106	mechanical and enzymatic digestion, FACS, nuclei isolation	Chromium Single Cells 3’ v2 or v3 (10x Genomics); Illumina NextSeq 500 or Hiseq 4000	adult human CM and non-myocyte nuclei (with selected upsampled whole cells)	Healthy human adult cardiac tissue: biopsies from four chambers plus septum and apex

## 7. Single-Cell Analysis and Implications for COVID-19

The global pandemic caused by SARS-CoV-2 has significantly impacted the quality of life for billions of people. The cardiovascular implications of the associated coronavirus disease 2019 (COVID-19) are threefold, as reviewed in [159,160,161,162]. First, although viral pneumonia is the most common clinical manifestation of COVID-19, the disease can instigate cardiac injury in diverse forms, including fulminant myocarditis [163,164,165], MI due to blood-clotting [166], and arrhythmia [167,168]. Additionally, the disease progression appears to be worse in elderly patients with pre-conditions such as diabetes, hypertension, and cardiac diseases [159]. Finally, SARS-CoV-2 utilizes the angiotensin-converting enzyme 2 (ACE2) as the primary entry receptor [169]. ACE2 is an essential component in the renin–angiotensin system (RAS) and is integral in proper blood pressure regulation. It limits the production of the vasoconstrictive AngII by cleaving Angiotensinogen to form Angiotensin 1–7 [170]. Circulating ACE2 is upregulated in different pathological conditions [171], and ACE2 activity seems to be increased in the heart upon treatment with commonly used angiotensin-converting enzyme inhibitors (ACEi) and angiotensin II receptor blockers (ARB) [172]. Given the potential for these anti-hypertensive therapies to increase entry receptors for the virus, their safety was initially questioned [170]. Fortunately, these concerns have been dissipated by clinical trials that showed no significant correlation between the use of these drugs and either the probability of contracting the virus or the severity of the disease [173].

Virus-associated pathologies are the result of complex host–pathogen interactions. scRNAseq could potentially be used to highlight cell-specific responses to viral infection in different tissues. To date, only one preprint manuscript reports the use of scRNAseq on infected human cells from the airway epithelium [174]. Bulk RNAseq has been adopted to profile hiPSC-CMs exposed to SARS-CoV-2 [175]. This study proved that CMs can be directly infected; upregulate genes related to immune response (cytokines such as *CXCL2*, antiviral genes such as *OAS3*) and apoptosis; and downregulate genes involved in oxidative phosphorylation, troponins, and the entry receptor *ACE2* (similar to previous observations in SARS-CoV-infected myocardium [176]). To our knowledge, no study has yet reported the transcriptomic changes occurring at a single cell level in infected cardiac organoid or tissue biopsies from infected or recovered patients. However, publicly available [177,178,179] and newly generated [135,154,180,181,182] single-cell datasets have been interrogated to identify the expression patterns of the entry receptor ACE2 and the proteases necessary for the priming of the viral S-protein (Table 7). These studies showed that hACE2 expression is highest in cardiac pericytes, followed by fibroblast and CMs. The expression of the TMPRRS2 protease is negligible in the heart, but other proteases such as CTSB, CTSL, and FURIN, all of which are expressed at a low level in nearly all cardiac cell types, could act in its place to facilitate virus uptake. The expression of ACE2 is upregulated with aging in non-human primates both in CMs and arterial ECs [135]. It is also upregulated in CMs from patients with heart failure, although there is debate on whether this increased ACE2 expression is restricted to CMs: one study showed a global upregulation of ACE2 in failing hearts at both the RNA and protein level [180], while two others showed that the increase in expression in CMs is compensated by a relative decrease in pericytes and other interstitial cell types [181,182]. In response to ACEi treatment, another study showed a significant upregulation of ACE2 in CMs [181], while a second reported increased expression in all cardiac cell types [182]. Finally, scRNAseq on circulating immune cells in patients with heart failure has shown an increase in monocyte/T cell ratio and dramatic transcriptional changes in the monocyte populations, indicating an enhanced pro-inflammatory state [127].

Overall, these data provide insight into cardiovascular susceptibility to SARS-CoV-2 infection. They suggest that pericytes may be the first port of entry for the virus in healthy hearts, leading to vascular damage and thrombosis, followed by entry via fibroblasts, which could contribute to worsening diastolic dysfunction. The susceptibility of elderly patients and patients with comorbidities to more severe myocardial injury may be due in part to elevated ACE2 expression and immune system dysregulations.

## 8. Current Challenges and Future Prospective

As single-cell analyses permeate both public and private research in single-cell analysis, the rapid advancement of novel experimental techniques and algorithmic innovations promises to lead to increasingly precise and efficient tools for genetics and cell biology. Innovation in scRNAseq technology is occurring at an unprecedented rate; multiple scRNAseq methods have already been developed [22], and the number of cells analyzed in cardiac scRNAseq studies is increasing exponentially (Figure 1B). The development of antibody-based cell hashing is enabling multiplexing by indexing samples, which can drive down costs and allow for more complex experimental designs [183]. As single-cell technology advances, it is poised to both become a more accessible tool for researchers and to provide highly resolved portraits of cardiac tissue functional state at homeostasis or in pathological contexts.

A limitation of high-throughput scRNAseq methods is that the data are sparse, with a large fraction of the gene counts equal to zero. Zero counts in scRNAseq can result from technical factors (mRNA is not captured) or can be biological in origin (the gene is not expressed in a particular cell) [184,185]. Several new approaches hold promise for partially ameliorating these limitations. The simultaneous detection of RNA and protein at the single-cell level [17,18] could reveal the presence of stable proteins with few or no cognate transcripts currently present in the cell. This method is particularly useful for precisely defining several immune cell subtypes that are traditionally classified using the expression of surface antigens. Quantitative readouts of chromatin accessibility using snATAC-seq can further support single-cell transcriptomic data [28,64,91]. To maximize the transcriptomic coverage for differential gene expression analysis, a recent study has combined cell-type information from scRNAseq with bulk RNAseq data from the same tissue using a deconvolution method [94].

Commonly used scRNAseq methods generate libraries that are amenable to high-throughput short-read sequencing by capturing the 3′ or 5′ end of each transcript, combined with a tag including the cellular barcode and a unique molecular identifier [8,9,10,11,12,13,14,15]. Short-read sequencing of end-primed transcripts hampers the detection of transcript isoforms, although some scRNAseq methods provide full-length transcript information [186,187]. Methods are currently being developed to combine short-read and long-read sequencing from barcoded cells to identify cell-type specific isoforms through barcode deconvolution [188].

A great advantage of scRNAseq is the ability to observe cells as they progress along a continuum of development, differentiation, or disease. Nevertheless, typical pseudo-temporal trajectories inferred from transcriptomic data lack directionality and may not reflect a real biological hierarchy. One novel algorithmic approach to this problem is RNA velocity [189], which harnesses reads derived from introns to assess transcriptional activity at present versus in the past (e.g., unprocessed compared to processed transcript abundances). Moreover, new experimental methods are allowing investigators to combine scRNAseq with clonal lineage tracing by introducing a DNA barcode [190] or CRISPR–Cas9-induced genetic modifications [191,192] in the parental cell.

A continual challenge in single cell biology is the confounding effect of cell isolation, which may introduce changes in gene expression. To compensate for this problem, transcriptomic tools are currently being optimized to enable the preservation of spatial information during RNA capture. There are already multiple platforms for in situ RNAseq, which can be broadly classified as either targeted or unbiased approaches, as reviewed in [193]. Targeted methods allow for the detection of a limited number of pre-defined targets at high resolution, while unbiased methods can in principle detect the full transcriptome but with limited resolution. One of the first unbiased methods was Spatial Transcriptomics [50], which has been acquired by 10X Genomics and is presently marketed under the Visium tradename. In this approach, tissue sections are placed on glass slides pre-printed with clusters of barcoded primers. The RNA is captured and retrotranscribed in situ, and the RNA–DNA complexes are collected for amplification and sequencing ex situ. The distance between the clusters of primers limits the resolution to regions, currently about 30um in size, that may include multiple cells. Currently, this approach has been used in two cardiovascular studies, which have sought to bypass limits on resolution by combining spatial transcriptomic data with scRNAseq through computational deconvolution methods in order to reconstitute detailed spatial transcriptomic maps [51,194]. In this fast-developing field, we consider it likely that imaginative new advances will eventually allow unbiased RNA and protein detection on the same tissue section with increasing resolution and sensitivity.

In light of the deluge of recent advances in single cell analysis, it is becoming essential to develop and optimize methods for the integration of data of different types and from different sources [195]. Single-cell isolation methods may vary dramatically between research groups, and this may introduce confounding variables for single-cell study integration. Recently, a series of guidelines on how to report scRNAseq experiments have been published to ensure reproducibility [196]. The standardization of public scRNAseq data submission will facilitate the future integration of many disparate single-cell datasets, which will be a valuable resource for hypothesis generation in cardiovascular biology.

## 9. Conclusions

Although relatively new, single-cell transcriptomics has already revealed numerous insights in cardiovascular biology. Single-cell analysis of cardiac development has developed our understanding of established key early cardiac transcription factors (Table 1). Single-cell computational trajectories have provided insight into the lineage commitment and differentiation of CPs in vitro (Table 2 and Table 3). Numerous cardiac single-cell atlases have been developed in multiple organisms and in multiple contexts including genetic variation, biological sex, and cardiac injury, all of which will provide useful resources for future work (Table 6). Characterizing injury responses in both regenerative and non-regenerative contexts has revealed previously undescribed intermediate cells as well as mechanisms of paracrine signaling, which contribute to regeneration and scar formation (Table 4 and Table 5). Disease-related cell-specific transcriptomic signatures can be used as diagnostic and prognostic tools. Most recently, single-cell analysis has provided useful information regarding the cardiac consequences of SARS-CoV-2 infection as multiple groups characterized the expression of the entry receptor hACE2 among cardiac cells (Table 7).

Taken together, single-cell analysis of the heart has revealed previously underappreciated cellular heterogeneity and the importance of paracrine intercellular communication. This diversity of cardiac cell types (and cell subtypes) acting in concert likely contributes to the homeostatic maintenance of cardiac tissue and is integral in the complex biological processes that govern progenitor cell differentiation, cardiovascular development, disease, and regeneration. The use of single-cell analytics will enable the definition of a healthy cardiac cell system and thereby better equip therapeutic pursuit toward the maintenance of this healthy cell system during physiological stress.

## Figures and Tables

**Table 7:** Cardiac Single-cell and COVID-19.

Authors PMID	Date	# of Cells and/or Nuclei	Isolation Method	Sequencing Technology	Target Cell Types	Context
Chen et al. [180] PMID: 32227090	March 2020	does not specify	enzymatic digestion, nuclei isolation	Chromium Single Cells 3’ v3 (10x Genomics); Illumina Hi-seq Xten	all human heart cell types	hACE2 expression in healthy and failing human hearts
Nicin et al. [181] PMID: 32293672	April 2020	57,601	enzymatic digestion, nuclei isolation	does not specify	all human heart cell types	hACE2 expression in healthy and failing human hearts (1 healthy, 5 aortic stenosis, 2 HFrEF patients)
Tucker and Chaffin et al. [182] PMID: 32795091	June 2020	677,785	mechanical and enzymatic digestion, nuclei isolation	Chromium Single Cells 3’ v3 (10x Genomics)	All human LV cell types	Healthy (n = 11) and failing adult human heart (11 dilated-, 15 hypertrophic- cardiomyopathy)

## Abbreviations

AP	Action potential
CFs	Cardiac fibroblasts
CMs	Cardiomyocytes
CPs	Cardiac progenitors
DCM	Dilated cardiomyopathy
E	Embryonic day
EB	Early allontoic bud stage
ECM	Extracellular matrix
ECs	Endothelial cells
EHF	Early headfold stage
ESCs	Embryonic stem cells
FHF	First heart field
HF	Heart failure
HFrEF	Heart failure with reduced ejection fraction
HLHS	Hypoplastic left heart syndrome
hpf	Hours post fertilization
I/R	Ischemia/reperfusion
iCM	Induced cardiomyocytes
IFC	Integrated fluidic circuits
imCM	Immature CM (p1 hearts)
iPSCs	Induced pluripotent stem cells
KO	Knock-out
LRHS	Hypoplastic right heart syndrome
LV	Left ventricle
MACS	Magnetic activated cell sorting
MI	Myocardial Infarction.
MSC	Mesenchymal stem cells
NSTEMI	Non-ST segment elevation myocardial infarction
OFT	Outflow tract
p	Postnatal day
ps	Pairs of somites
RNAseq	Ribonucleic acid sequencing
ROS	Reactive oxygen species
RV	Right ventricle
SHF	Second heart field
SMCs	Smooth muscle cells
SV	Sinus venosus
TAC	Trans aortic constriction
UMI	Unique molecular identifier
wt	Wild type
